# Endoscopic Resection for Small Rectal Neuroendocrine Tumors: Comparison of Endoscopic Submucosal Resection with Band Ligation and Endoscopic Submucosal Dissection

**DOI:** 10.1155/2016/6198927

**Published:** 2016-07-25

**Authors:** Byoung Wook Bang, Jin Seok Park, Hyung Kil Kim, Yong Woon Shin, Kye Sook Kwon, Joon Mee Kim

**Affiliations:** ^1^Division of Gastroenterology, Department of Internal Medicine, Inha University School of Medicine, Incheon 22332, Republic of Korea; ^2^Division of Gastroenterology, Department of Pathology, Inha University School of Medicine, Incheon 22332, Republic of Korea

## Abstract

*Background and Aims*. There is no consensus so far regarding the optimal endoscopic method for treatment of small rectal neuroendocrine tumor (NET). The aim of this study was to compare treatment efficacy, safety, and procedure time between endoscopic submucosal resection with band ligation (ESMR-L) and endoscopic submucosal dissection (ESD).* Methods*. We conducted a prospective study of patients who visited Inha University Hospital for endoscopic resection of rectal NET (≦10 mm). Pathological complete resection rate, procedure time, and complications were evaluated.* Results*. A total of 77 patients were treated by ESMR-L (*n* = 53) or ESD (*n* = 24). En bloc resection was achieved in all patients. A significantly higher pathological complete resection rate was observed in the ESMR-L group (53/53, 100%) than in the ESD group (13/24, 54.2%) (*P* = 0.000). The procedure time of ESD (17.9 ± 9.1 min) was significantly longer compared to that of ESMR-L (5.3 ± 2.8 min) (*P* = 0.000).* Conclusions*. Considering the clinical efficacy, technical difficulty, and procedure time, the ESMR-L method should be considered as the first-line therapy for the small rectal NET (≤10 mm). ESD should be left as a second-line treatment for the fibrotic lesion which could not be removed using the ESMR-L method.

## 1. Introduction

Rectal NETs are rare tumors, most of which are diagnosed incidentally during routine colonoscopy. Rectal NETs are epithelial tumors that arise from the deep portion of glands. They typically invade through the muscularis mucosa into the submucosa and therefore resemble subepithelial tumors [1]. In Korea, in particular, rectal NET is the most common (48%) among all gastrointestinal NETs [2] and its incidence has shown a remarkable increase in recent decades [2–4]. In Korea, screening colonoscopy is recommended from the age of 50 in the average-risk group and follow-up colonoscopy is recommended every 5 years when index colonoscopy is negative [5]. Because of such a colon cancer screening program, the chance of encountering small rectal NET (≤10 mm) has increased. Approximately 80% of rectal NETs are 10 mm or less in size and contained within the submucosal layer; therefore, they are suitable for endoscopic removal [3, 6, 7].

Various endoscopic techniques have been developed including endoscopic mucosal resection (EMR), ESD, and ESMR-L. Conventional EMR has been commonly performed for treatment of colonic polyps; however, due to its high incomplete resection rate (14% to 62%), it is considered inadequate for treatment of rectal NET [8, 9]. The ESD method for rectal NET has recently been regarded as a valuable endoscopic treatment, because it provides a higher en bloc resection rate than conventional EMR, enabling accurate pathologic diagnoses [10, 11]. However, the ESD technique requires a high level of endoscopic experience and takes more time to perform. In addition, NET usually involves the submucosal layer; thus, achievement of a sufficiently deep resection margin might be difficult by ESD technique. ESMR-L has also been reported to be an effective and safe procedure for treatment of small rectal NET (≤10 mm) [12]. In the ESMR-L method, the tumor is ligated with a band and a snare is placed below the band for resection, enabling achievement of a deeper resection plane. However, there is still debate regarding the best endoscopic treatment for small rectal NET (≤10 mm). Several meta-analysis studies have reported that ESD is more effective than EMR regarding complete resection [13, 14]. However, few studies comparing ESD and ESMR-L have been reported [15].

The aim of this study was to prospectively compare complete resection rate, safety, and procedure time between ESMR-L and ESD technique for treatment of small rectal NET (≤10 mm).

## 2. Methods

### 2.1. Patients and Lesions

This study was conducted in patients with small rectal NET (≤10 mm) who underwent endoscopic resection at Inha University Hospital between January 2012 and February 2016. Endoscopic ultrasound (EUS) was performed before the endoscopic resection to evaluate the exact size of the tumor and to determine submucosal space and proper muscle involvement. Pretreatment abdominal computed tomography (CT) was performed in all patients to rule out local and distant metastasis. Indications of endoscopic treatment were as follows: ≤10 mm in diameter and confined to the submucosal layer as assessed by EUS (GF-UC240P-AL5, Olympus Optical Co., Tokyo, Japan). The current study was approved by the institutional review board of Inha University Hospital, Incheon, Korea (IRB number 13-015).

### 2.2. Endoscopic Procedures

Endoscopic procedures were performed by four endoscopists with ESD experience of more than five years (Hyung Kil Kim, Yong Woon Shin, Kye Sook Kwon, and Byoung Wook Bang). Bowel preparation with a polyethylene-glycol solution and ascorbic acid (Coolprep®, TaeJoon Pharmaceuticals, Seoul, Korea) was performed before endoscopic treatment. Informed consent was obtained from all patients before endoscopic treatment.

ESMR-L technique was as follows. First, to elevate the tumor, a mixture of saline solution was injected into the submucosal layer beneath the tumor. The lesion was aspirated into the transparent cap, followed by deployment of the elastic band. A polypectomy snare (Olympus Optical Co., Tokyo, Japan) was then used to perform a ligation below the elastic band, and resection was performed above the band using cutting current, generated using a VIO300D electrosurgical unit (ERBE, Tuebingen, Germany). When the lesion was not sucked into the cap, ESD was performed as rescue treatment.

For ESD, saline solution mixed with indigo carmine was injected into the submucosal layer around the lesion. A circumferential incision was made at 3–5 mm outside the tumor using a Dual Knife (KD-650Q, Olympus Optical Co., Tokyo, Japan). After an additional injection of saline solution beneath the lesion, the submucosal layer was directly dissected using the same knife.

Resected specimens were evaluated histologically using light microscopy to determine histological type, depth of invasion, lateral and vertical resection margin involvement, and lymphovascular invasion. Immunohistochemical staining for neuron-specific enolase and synaptophysin was performed to support the diagnosis. Mitotic count and Ki-67 index were assessed for determination of tumor grade. En bloc resection was defined as resection of the entire lesion in a single piece. Pathological complete resection was defined as no involvement of tumor cells on the lateral and vertical margin of the resected tumor on microscopy. Procedure time was defined as the period from identification of the lesion to completion of the resection and bleeding control. Significant delayed bleeding was defined as hematochezia after completion of endoscopic resection that required hemostasis or transfusions. Perforation was defined as rectal wall penetration observed during the endoscopic procedure or detected after endoscopy by radiologic examination such as abdominal CT.

### 2.3. Statistical Analyses

Statistical analyses were performed using SPSS software (version 18.0; SPSS Chicago, IL, USA). Student's *t*-test and chi-square test were used for comparison of continuous and categorical data between the two groups. *P* values < 0.05 for two-tailed tests were considered significant.

## 3. Results

A total of 77 patients were enrolled in this study (53 ESMR-L cases and 24 ESD cases). In two cases, we tried to perform ESMR-L; however, the lesions were not sucked into the cap because of previous biopsy induced scar change. Therefore, we performed ESD and included them in the ESD group. Mean age of patients was 52.7 ± 12.6 years. The mean diameter of resected tumors between ESMR-L and ESD was 4.6 mm versus 5.2 mm (*P* = 0.137), respectively (Table 1). All patients were asymptomatic and none had symptoms or signs of carcinoid syndrome. The mean procedure time was 5.3 ± 2.8 minutes and 17.9 ± 9.1 minutes in the ESMR-L group and ESD group, respectively (*P* = 0.000) (Table 2).

**Figure 1:**
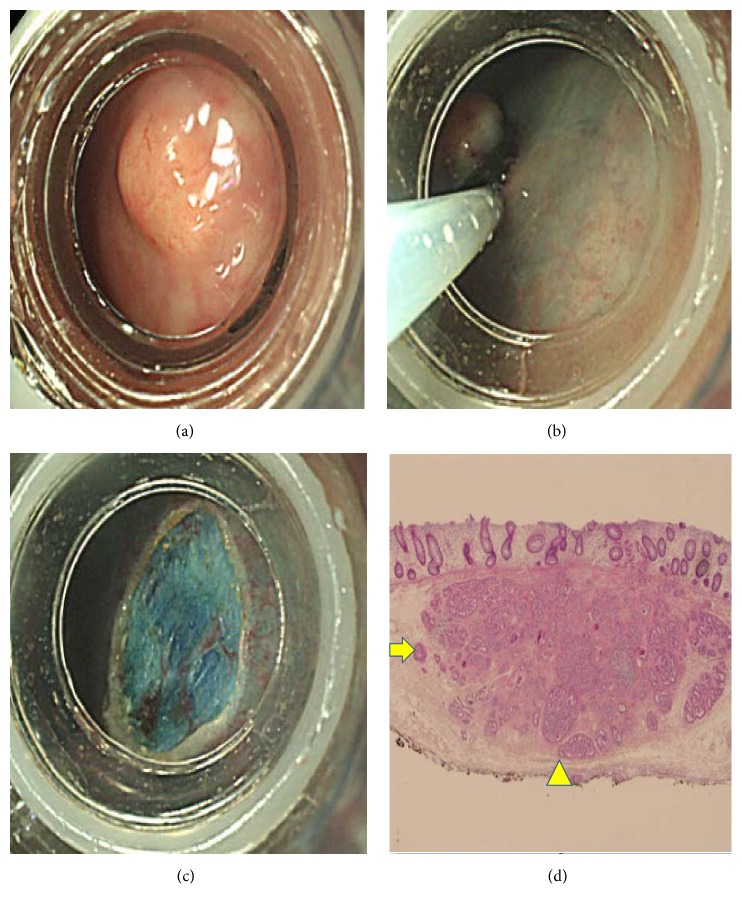
Endoscopic submucosal resection with band ligation. A yellowish tumor was detected (a) and band ligation was performed (b) followed by snare polypectomy (c). The tumor was completely resected (d). Pathologic finding showed a negative lateral (arrow) and deep (arrowhead) resection margin.

**Figure 2:**
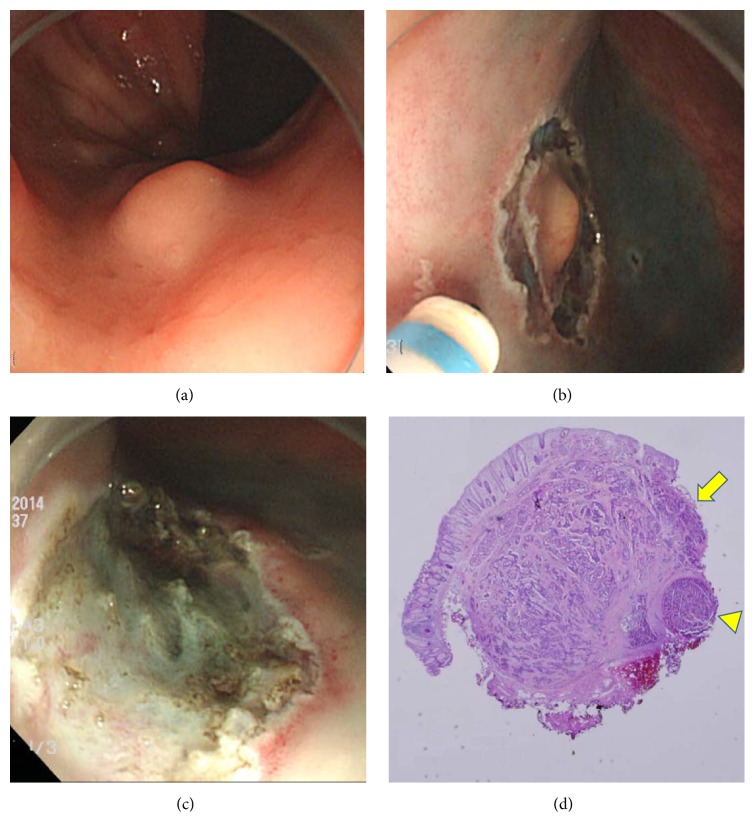
Endoscopic submucosal dissection. A yellowish tumor was detected in the rectum (a). After circumferential mucosal incision (b), ESD was performed and gross endoscopic complete resection was achieved. (c) However, pathological finding showed deep (arrowhead) and lateral (arrow) resection margin involvement (d).

Gross endoscopic complete resection was achieved in all patients. Regarding the histologic examination, complete resection was achieved in 100% (53/53) of patients in the ESMR-L group (Figure 1). However, only 54.2% (13/24) of patients in the ESD group showed complete resection histologically (Figure 2) (*P* = 0.000). Among 11 patients with histologic incomplete resection, deep, lateral, and both resection margins were positive in seven, one, and three patients, respectively. Therefore, 41.7% (10/24) of patients in the ESD group showed deep resection margin involvement. All patients with histologic incomplete resection underwent follow-up endoscopy for a short-term period. However, no remnant tumor was found; thus, careful follow-up was performed without additional resection. Lymphovascular invasion was observed in one and two patients in the ESMR-L and ESD group, respectively. However, additional surgery with lymph node dissection was not performed because of the patients' decision. They were carefully followed up without recurrence. In the ESMR-L group, there were two cases of postprocedure bleeding, who were treated with coagulation therapy. One patient developed a new rectal metachronous NET after one year of follow-up, which was also resected using the ESD technique. No distant metastasis was observed at the last follow-up examinations in all patients.

## 4. Discussion

Treatment of rectal NET depends mainly on size, which is the simplest way of predicting tumor behavior. Small rectal NET (≤10 mm) confined to the submucosal layer without lymphovascular invasion is considered a good candidate for endoscopic resection [7]. In addition, intensive surveillance imaging and endoscopic examination after endoscopic resection are not recommended because of the low risk of metastasis [16]. According to ENETS consensus, endoscopic treatment is regarded as the appropriate therapy for small rectal NET (≤10 mm) [7].

Optimal endoscopic techniques have not been determined. EMR has been used because of its simplicity and less invasiveness. However, because NETs are mainly located in the submucosal layer of the rectal wall, achievement of pathological complete resection is difficult using this technique. ESD technique was originally developed for early gastric cancer in the stomach. The ESD technique was recently applied for treatment of rectal NET [10, 17]. In previous studies, nearly 100% negative lateral resection margin was achieved using the ESD technique, while vertical resection involvement of 6.5% to 19.4% was reported [10, 15, 17]. Theoretically, sufficient lateral margin is feasible using ESD; however, obtaining a sufficient vertical margin is not easy due to infiltration of rectal NET into the submucosal layer.

Several articles regarding ESD for rectal NET published from 2010 reported excellent results [10, 17]. From 2012, we have prospectively performed ESD for treatment of rectal NET. In the current study, 45.8% of patients treated with ESD showed histologic incomplete resection, which was markedly higher than in previous studies [10, 15, 17]. One reason was thought to be inappropriate specimen preparation. We did not stretch the resected specimen laterally using a pin because specimens were too small to fix, which could cause overdiagnosis of involvement of lateral resection margin during tissue processing. In addition, dissection of small lesions was difficult using the ESD technique because it was not separated from the mucosa. Lack of ESD experience of rectal NET might be one reason. However, endoscopists had more than 5 years of therapeutic endoscopic experience and each of them had performed 50 to 300 colonic ESD before starting this study. In the current study, ESD required more time to perform and carried a higher risk of incomplete histologic resection than we expected [10, 13].

After disappointing results, we have performed ESMR-L instead of ESD for treatment of small rectal NET (≤10 mm). It was technically easier and faster to perform than ESD with 100% histological complete resection rate. However, two NETs were not aspirated into the cap because of previous biopsy induced severe scar change. For those lesions, ESD was a useful treatment method. There were two cases of delayed bleeding after ESMR-L. Therefore, routine prophylactic coagulation of visible vessels after ESMR-L might be required to prevent delayed bleeding.

Although 45.8% of patients in the current study who were treated with ESD showed incomplete resection histologically, none of the patients received additional treatment. We considered them clinically complete resection because of cautery effect of the resected plane and pseudocapsule formation around the tumor mass [18]. In addition, we followed up three patients with lymphatic invasion without additional surgery. Although controversial, a recent study reported excellent long-term prognosis following endoscopic resection of patients with rectal NET despite lymphovascular invasion [19].

The current study demonstrated the superiority of ESMR-L in the viewpoint of histological complete resection and procedure time. ESMR-L offers some advantages in the treatment of rectal NET. First, ESMR-L is more effective for deep resection than ESD. Suctioning the rectal wall using a transparent cap can aspirate deep submucosal layer into the cap; therefore, ESMR-L provides a deeper vertical resection margin. In addition, compared with the colon, the rectum is encircled by a relatively thick muscle layer; therefore, suctioning of a submucosal tumor involves little chance of perforation. Second, ESMR-L is suitable for rectal NET which usually appears as a small, round, and submucosal nodule with a well-demarcated tumor margin. Unlike lateral spreading tumor, a broad free lateral margin is not necessary for rectal NET. Third, ESMR-L is relatively simple, easy, and less time-consuming compared with the ESD procedure, which requires significant endoscopic experience and skill. In addition, ESMR-L is economically advantageous because it does not require use of various endoscopic knives.

There were several limitations to our study. First, this study was a nonrandomized study conducted in a single center. Patients were assigned to the ESD group in the early period of this study. After changing endoscopic method, most patients were assigned to the ESMR-L group except two cases with fibrotic lesions. Second, the follow-up duration was relatively short. However, our main objective was to assess short-term results including procedure time and pathological resection rate. Despite these limitations, our study demonstrated that the ESMR-L technique is superior to ESD for treatment of the small rectal NET (≤10 mm) and ESD is not suitable as a first-line treatment for small rectal NET (≤10 mm).

In conclusion, based on the results of this study, ESMR-L should be considered as the first-line therapy for the small rectal NET (≤10 mm). ESD may be suboptimal for small rectal NET and it should be left as a second-line therapy when ESMR-L is not applicable. However, conduct of a randomized controlled study will be needed in order to confirm our results.

## Figures and Tables

**Table 1:** Patients and tumor characteristics for the ESMR-L and ESD groups.

	ESMR-L (*n* = 53)	ESD (*n* = 24)	Total (*n* = 77)	*P* value
Age (year)	53.6 ± 12.7	50.8 ± 12.4	52.7 ± 12.6	0.378
Sex (M/F), *n*	33/21	21/6	50/27	0.311
Tumor size (mm)				
EUS^‡^	5.0 ± 1.7	5.5 ± 2.1	5.2 ± 1.9	0.220
Histology^‡^	4.6 ± 1.7	5.2 ± 1.9	4.7 ± 1.8	0.137
Distance from AV^†^ (cm)	5.5 ± 2.8	5.5 ± 2.5	5.5 ± 2.7	0.895

EUS: endoscopic ultrasound.

^†^AV: anal verge.

^‡^Values are expressed as mean ± SD.

**Table 2:** Endoscopic outcomes and follow-up of endoscopic submucosal resection with band ligation (ESMR-L) and endoscopic submucosal dissection (ESD) groups.

	ESMR-L (*n* = 53) number (%)	ESD (*n* = 24) number (%)	*P* value
En bloc resection	53 (100.0)	24 (100.0)	NS
Histological complete resection	53 (100.0)	13 (54.2)	0.000
Histological margin involvement	0 (0)	11 (45.8)	0.000
Deep	0 (0)	7 (29.2)	
Lateral	0 (0)	1 (4.2)	
Both	0 (0)	3 (12.5)	
Lymphatic invasion	1 (1.9)	2 (8.3)	0.228
WHO grade 1/2/3	52/1/0	21/3/0	0.052
Procedure time (min)^‡^	5.3 ± 2.8	17.9 ± 9.1	0.000
Perforation	0 (0)	0 (0)	NS^†^
Bleeding	2 (3.8)	0 (0)	0.335
Follow-up (month)^‡^	7.8 ± 11.2	22.3 ± 16.8	0.001
Metachronous lesion or recurrence	0 (0)	1 (4.2)	0.312
Distant metastasis	0 (0)	0 (0)	NS^†^

^†^NS: not significant.

^‡^Values are expressed as mean ± SD.
